# Strengthening TikTok Content Analysis in Academia Using Follower Count and Engagement

**DOI:** 10.2196/54439

**Published:** 2024-01-30

**Authors:** Serena Ramjee, Zeeshaan-ul Hasan

**Affiliations:** 1 Urology Department Darent Valley Hospital Dartford United Kingdom; 2 Dermatology Department Barts Health NHS Trust London United Kingdom

**Keywords:** social media, skin of color, skin of colour, representation, TikTok, atopic dermatitis, dermatology, dermatologist

## Letter

We read with great interest Abdelnour et al’s paper titled “Skin of color representation for atopic dermatitis on TikTok: cross-sectional analysis” [1] and express our gratitude for the findings.

Using the search term #eczema in July 2022, the study evaluated the representation of patients with skin of color (SoC) and the quality of atopic dermatitis videos on TikTok. A review of 119 eligible videos revealed that physicians produced significantly higher-quality content than nonphysicians but may underrepresent SoC. Viewer count was a secondary measure, with its mean value lower for physicians compared to nonphysicians, though the difference was not significant. The authors noted that this lower viewer count may limit the impact of better SoC representation in physicians’ videos. However, we believe that this conclusion cannot be made without further analysis.

Using the viewer count, one may infer that physician content is less popular. However, in instances where there is an insignificant difference in viewer count between sources, this measure alone provides limited information. On TikTok, a view is “counted” within the 3 seconds of playback, meaning a user does not have to view the entire video. Additionally, the viewer count corresponds to the number of times a video has been played rather than unique views [2]. These factors, coupled with TikTok automatically replaying its videos once they finish, mean the viewer count does not reflect the number of individuals that have viewed a video.

Follower count and engagement (likes, saves, shares, and comments) provide additional context. These measures, alongside viewer count, enable the calculation of a video’s engagement rate and reach percentage (view rate). Engagement rate estimates the percentage of viewers that engage with a video (engagement×100/viewer count) [3], whereas reach percentage estimates the percentage of a source’s followers that view a video (viewer count×100/followers) [2]. Marketing companies suggest a “good” engagement rate lies between 1% to 5% [3] and define the average reach percentage as 14.49% [2]. To demonstrate the application of these formulas, we reviewed the results from Pagani et al [4] below.

This cross-sectional study screened the top 50 videos when searching “slugging” (defined as thickly coating the skin with a petrolatum-based ointment like Vaseline and can form the final step of a nighttime skincare routine [4]) on TikTok and analyzed their upload source, content, and quality. Videos were categorized by source into health care providers, influencers, and others. Assessing follower count and engagement (likes and comments) revealed that although influencers have a nonsignificantly lower median viewer count than health care providers (94,500 vs 102,150), their videos had a greater reach percentage (65.3% vs 24.9%) and engagement rate (8.1% vs 4.3%). These values suggest that influencers created more engaging content, which may be better promoted by TikTok’s algorithm and result in a higher viewer count long term.

We observe that TikTok content analysis is becoming a prevailing means of understanding public dermatology-related information, an unsurprising trend since the platform’s video-based format favors dermatology’s visual nature, and believe follower count and engagement aid this analysis. Regarding the work of Abdelnour et al [1], these measures may assist in determining the impact of improved SoC representation in physician-produced atopic dermatitis videos. If these measures are low, targeted recommendations for improving engagement and reach can be suggested, such as integrating popular trends or cross-promoting content.

## Abbreviations

SoC: skin of color
